# Current Treatment Options for Renal Cell Carcinoma: Focus on Cell-Based Immunotherapy

**DOI:** 10.3390/cancers16061209

**Published:** 2024-03-19

**Authors:** Angela Hwang, Vedika Mehra, Jyoti Chhetri, Samira Ali, Maxine Tran, Claire Roddie

**Affiliations:** 1 Department of Haematology, University College Hospital, University College London Hospitals NHS Foundation Trust, London NW1 2BU, UK; angela.hwang@nhs.net; 2 University College London Cancer Institute, 72 Huntley Street, London WC1E 6DD, UK; vedika.mehra.15@ucl.ac.uk (V.M.); j.chhetri@ucl.ac.uk (J.C.); samira.ali@ucl.ac.uk (S.A.); 3 University College London Division of Surgery and Interventional Science, Rowland Street, London NW3 2QG, UK; m.tran@ucl.ac.uk; 4 The Specialist Centre for Kidney Cancer, Royal Free Hospital, Pond Street, London NW3 2QG, UK

**Keywords:** renal cell carcinoma, immunotherapy, chimeric antigen receptor (CAR) T-cells, engineered TCR T-cells

## Abstract

**Simple Summary:**

Renal cell carcinoma (RCC) is common, affecting over 400,000 patients globally each year. In limited stage disease and where surgery is a feasible therapeutic option, event free survival rates are high. In contrast, locally advanced disease is associated with frequent relapse, and up to 30% of patients present with metastatic disease. Standard of care treatments for metastatic disease include tyrosine kinase inhibitors (TKI) and immune checkpoint inhibitors (ICI), but clinical responses are variable and generally not durable. Novel cell-based immunotherapy approaches such as Chimeric Antigen Receptor T-cells (CAR-T) and T-cell receptor engineered T-cells (TCR-T) have shown efficacy in some blood cancers and are currently under evaluation in solid tumours including RCC. Here, we review the landscape of cellular immunotherapy for RCC in the context of currently available therapies and outline how advanced engineering solutions may further enhance this therapeutic approach.

**Abstract:**

Renal cell carcinoma (RCC) affects over 400,000 patients globally each year, and 30% of patients present with metastatic disease. Current standard of care therapy for metastatic RCC involve TKIs and ICIs, including combinatorial strategies, but this offers only modest clinical benefit. Novel treatment approaches are warranted, and cell-based immunotherapies for RCC hold significant promise. These are currently being tested in the pre-clinical setting and in early phase clinical trials. Here, we review the landscape of cellular immunotherapy for RCC in the context of currently available therapies, with a particular focus on defining the current best antigenic targets, the range of cell therapy products being explored in RCC, and how advanced engineering solutions may further enhance these therapies in the RCC space.

## 1. Introduction

### 1.1. Demographics

Kidney cancer remains a common cancer globally, with an estimated 431,288 new cases in 2020 worldwide [1]. It remains within the top ten leading cancers in the United States, accounting for 5% of new cancer diagnoses in men and 3% in women [2]. Similarly, in the United Kingdom, kidney cancer is the 7th most common malignancy and accounts for 4% of new cancer diagnoses in men and 3% in women [3]. Despite improvements in diagnosis and treatment advancements, mortality rates have remained relatively static over the last decade and account for 2–3% of total cancer deaths [3,4].

Renal cell carcinoma (RCC) accounts for the majority of kidney cancers, with clear cell being the most common subtype in 75–85% of cases, followed by papillary, oncocytic and chromophobe subtypes [5]. Other renal tumours include much rarer and diverse tumours such as mucinous tubular and spindle cell carcinoma, and eosinophilic solid and cystic RCC. The updated 5th WHO classification of Tumours of the Urinary System and Male Genital Organs has also recently introduced a molecularly defined group of renal tumours including TFE3-rearranged RCC, TFEB-altered RCC, ELOC (formerly TCEB1)-mutated RCC and SMARCB1-deficient medullary RCCs, signalling a transition to a novel integrated diagnostic approach rather than morphology-based diagnostics [6]. Here, we will focus predominantly on the commonest subtype, i.e., clear cell RCC (ccRCC).

### 1.2. Staging

RCC staging is currently based on the Tumour, Nodes, Metastases (TNM) system which has undergone revision over the years. Based on current TNM criteria, T1 tumours are defined as < 7 cm, T2 tumours > 7 cm, T3 tumours involve major veins or perinephric tissue, not beyond Gerota’s fascia, whilst T4 tumours invade beyond Gerota’s fascia [7].

### 1.3. Risk Factors

The risk of RCC increases with age, with peak incidence between 60 and 70 years, and is higher in men with a 1.51:1 male predominance [8]. Further risk factors can be divided in hereditary or lifestyle factors and include chronic kidney disease, smoking, obesity, hypertension and inheritance of the rare familial von Hippel–Lindau (VHL) disease [9].

### 1.4. Molecular Pathology

Loss of chromosome 3p, the location of the VHL tumour suppressor gene, has been identified as an early event in the pathogenesis of RCC [10], and biallelic inactivation is seen in up to 90% of cases. The VHL protein is involved in ubiquitination and proteolysis of multiple intracellular targets such as hypoxia-inducible factor (HIF) proteins. Loss of VHL results in upregulation of HIF proteins and activation of HIF-related target genes such as vascular endothelial growth factor (VEGF). This induces a pseudo-hypoxic state and promotes angiogenesis [11]. Further, whole exome and genome sequencing studies have demonstrated loss of other tumour suppressor genes on chromosome 3p that contribute to RCC development, namely PBRM1, SETD2, BAP1 and TCEB1 [12].

### 1.5. RCC Immunogenicity

Research has demonstrated ccRCC to be an immunogenic cancer driven by a distinct tumour-specific antigen (TSA) profile. TSAs are uniquely expressed on tumours (and not normal cells), and as such are seen by the immune system as ‘foreign’ and can elicit immune activation. ccRCCs have a high prevalence of insertions and/or deletions resulting in frameshift mutations that can lead to the emergence of tumour-associated neoantigens that can trigger an immune response [13]. Human endogenous retroviruses (ERVs) are human genome elements that have accumulated through evolution, and when unsilenced, abnormal ERV expression can lead to the emergence of TSAs [14] in ccRCC [13]. ERV type E (ERV-E4) is specific to ccRCC and expression correlates with HIF2A activity. The ERV-K family has also been described in association with ccRCC [15].

### 1.6. RCC Tumour Microenvironment (TME)

The ccRCC TME is heterogeneous and unique [16,17], possessing a dense immune cell infiltrate comprising distinct CD8+ and CD4+ T-cell populations (tumour infiltrating lymphocytes (TILs)), with variable expression of activation and costimulatory markers compared to other RCC subtypes. In ccRCC, increased CD8+ T-cell infiltration has paradoxically been associated with poorer survival, which may reflect a terminally exhausted T-cell phenotype in advanced/metastatic disease, with less T-cell receptor (TCR) diversity [17]. The heterogeneity of tertiary lymphoid structures (TLS) in RCC has also been postulated to negatively impact the maturation of dendritic cells, leading to ineffective antigen presentation and the emergence of polyclonal CD8+ T-cell populations that do not recognise tumour-associated antigens [18]. Further within the ccRCC myeloid compartment, there is an abundance of immunosuppressive M2-like tumour-associated macrophages (TAMS) and myeloid-derived suppressor cells (MDSCs) that correlate with poor prognosis [19]. The complex, heterogeneous cellular environment in RCC is the subject of ongoing study.

## 2. Drug Treatments

### 2.1. Treatment for Localised ccRCC

For localised disease, surgery remains the only curative treatment [20]. However, up to one third of patients with locally advanced disease relapse following surgery with metastatic disease (mRCC) and will require further treatment [21,22]. Adjuvant therapy has been explored to improve outcomes although its role remains unclear. The phase 3 KEYNOTE-564 study randomised adjuvant Pembrolizumab, an anti-programmed death 1 (PD-1) antibody, versus placebo following nephrectomy +/ metastasectomy in patients with intermediate- to high-risk disease, and found a significant disease-free survival benefit (77.3% vs. 68.1% at 24 months, *p* = 0.002) [23]. This benefit however, has not been observed in other adjuvant immune checkpoint inhibitor (ICI) studies; this includes the Checkmate 914 trial that randomised combination adjuvant PD-1 inhibitor Nivolumab and cytotoxic T-lymphocyte antigen 4 (CTLA-4) inhibitor Ipilimumab versus placebo, the IMmotion010 that randomised adjuvant programmed death-ligand 1 (PD-L1) inhibitor Atezolizumab versus placebo, and the PROSPER study that compared neoadjuvant and adjuvant Nivolumab versus surgery alone [24,25,26].

### 2.2. Tyrosine Kinase Inhibitor (TKI)-Based Treatment for mRCC in the First Line

**Figure 1:**
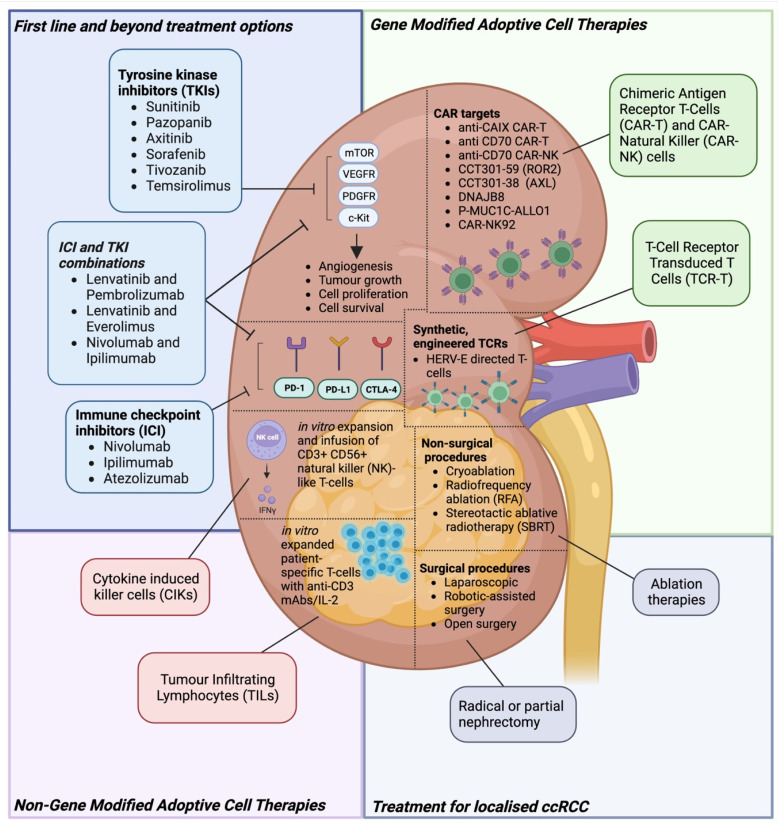
**Overview of Treatment Modalities for clear cell RCC (ccRCC).** The figure provides a comprehensive overview of the diverse treatment modalities employed for ccRCC. Surgical interventions and radical or partial nephrectomy employ various approaches for direct kidney or tumour removal. Tyrosine kinase inhibitors (TKIs) such as Sunitinib, Pazopanib and Axitinib disrupt critical signalling pathways associated with angiogenesis and cell proliferation. Immunotherapeutic agents, immune checkpoint inhibitors (ICIs) such as Nivolumab and Ipilimumab, enhance the immune response against cancer cells. Additionally, cytokine-induced killer cells (CIKs) and tumour-infiltrating lymphocytes (TILs) involve the selection and in vitro expansion of tumour-reactive cells, infused back into the patient for enhanced cytotoxicity. The emerging field of gene-modified adoptive cell therapies for ccRCC, specifically focusing on Chimeric Antigen Receptor T-Cells (CAR-T) and CAR-NK cells, is highlighted. This includes promising CAR-T therapies targeting CAIX, CD70, AXL, ROR2, and DNAJB8, with ongoing phase I/II clinical trials representing advancements in this area. Additionally, engineered T-cell receptors (TCRs), exemplified by HERV-E-directed TCR-T therapy, are designed to recognize intracellular tumour-associated antigens, presenting a promising avenue for personalized treatment in ccRCC.

Management of mRCC has evolved significantly over recent years (Figure 1). Historically, mRCC was treated with cytokine-based immunotherapies such as high-dose interleukin 2 (IL2) and interferon alpha (IFNα), but only a minority of patients derived long term benefit, and treatment was associated with severe toxicity [27]. Tyrosine kinase inhibitors (TKIs) represent an alternative treatment approach.

In a phase 3 study of 750 patients with newly diagnosed mRCC, Sunitinib, a multitargeting TKI of vascular endothelial growth factor receptor (VEGFR) and platelet-derived growth factor receptor (PDGFR), improved progression free survival (PFS) over IFNα alone (median 11 months vs. 5 months, *p* < 0.001) with improved OS (median 26.4 vs. 21.8 months, *p* = 0.051) [28] and was approved by the FDA in 2006. Similarly, a phase 3 study of Pazopanib, an oral angiogenesis inhibitor targeting VEGFR, PDGFR and c-KIT, in 435 ccRCC patients (233, treatment-naïve) improved PFS over placebo (median 11.1 vs. 2.8 months, *p* < 0.0001) and was FDA approved in 2009 [29].

Sorafenib is a TKI targeting VEGFR, PDGFR and c-KIT [30] and has been tested in the first line in 189 treatment-naïve patients vs. IFNα [31], but the median PFS was similar between treatment arms (5.7 vs. 5.6 months). Beyond first-line, Sorafenib vs. placebo was tested in a large pivotal phase 3 randomised study of 903 relapsed/refractory patients. Whilst the median PFS was significantly longer with sorafenib (5.5 vs. 2.8 months, *p* < 0.01), a significant OS benefit was not observed [32].

A trial of Bevacizumab, a monoclonal antibody against VEGF, in combination with IFNα-2a compared to IFNα-2a alone improved median PFS from 5.4 months to 10.2 months (*p* = 0.0001) [33]. Other agents in this space include Temsirolimus, an inhibitor of mammalian target of rapamycin (mTOR) kinase. Compared to IFNα monotherapy and in combination, Temsirolimus alone improved OS (median 10.9 vs. 7.3 months, *p* = 0.008), but in combination did not demonstrate PFS or OS benefit [34]. Tivozanib, a potent and selective VEGF inhibiting TKI, showed improved PFS (11.9 vs. 9.1 months, *p* = 0.042) but not OS (median 29.3 vs. 28.8 months, *p* = 0.105) [35]. The randomised phase 2 CABOSUN study compared the novel multi-targeting TKI Cabozantinib (VEGF, MET proto-oncogene-encoded receptor tyrosine kinase (MET) and AXL receptor tyrosine kinase (AXL)) with Sunitinib as first line therapy in intermediate/poor risk RCC. Cabozantinib demonstrated a significant PFS benefit (8.6 months vs. 5.3 months, *p* = 0.0008) [36]. In summary, whilst there has been much activity in the drug development space in ccRCC, there have been no recent paradigm-changing agents emerging in the front-line therapy setting.

### 2.3. TKI-Based Treatment for mRCC beyond 1st Line

Drug resistance to targeted molecular therapies is a major cause of treatment failure in ccRCC, and sequential administration schedules of novel/alternative TKIs as a strategy to overcome this have been tested in multiple clinical trials. The AXIS study of Axitinib, a second generation selective TKI versus Sorafenib in second line showed a modest PFS benefit (6.7 vs. 4.7 months, *p* < 0.0001) [37]. A phase 3 study of the mTOR inhibitor Everolimus vs. placebo in patients progressing post-TKI showed longer median PFS of 4.0 vs. 1.9 months [38]. A phase 2 study of Lenvatinib, a TKI targeting VEGF and fibroblast growth factor receptor 1 (FGFR1), vs. Everolimus monotherapy vs. combination therapy favoured combination, with improved PFS (median 14.6 vs. 5.5 months, *p* = 0.0005) [38,39]. More recently, a phase 3 study further demonstrated Cabozantinib improved OS and PFS compared to Everolimus alone [40].

### 2.4. Immune Checkpoint Inhibitors (ICI) for mRCC in the 1st Line and beyond

ICIs have transformed the therapeutic landscape in relapsed/refractory (r/r) mRCC and have now, on the whole, superseded TKIs as first line therapy. The CheckMate 025 study of Nivolumab vs. Everolimus in r/r patients demonstrated an OS benefit (25 vs. 19.6 months, HR 0.73) [41]. In the first line setting, the phase 3 CheckMate 214 trial of combinatorial Nivolumab and Ipilimumab improved ORR and OS compared with single agent Sunitinib, with the greatest benefit in those with ≥1% PD-1 expression [42]. Long term follow up (≥4 years) showed that combination ICI led to prolonged OS in both the ITT group (not reached (NR) vs. 38.4 months, HR 0.69) and the intermediate/poor risk group (48.1 vs. 26.6 months, HR 0.65) [43]. Median OS was NR in either group for those with favourable risk disease. With clearly superior treatment outcomes compared to previous TKI therapy, these studies established ICIs as standard of care in mRCC [20].

### 2.5. ICI and TKI Combinations for mRCC in the 1st Line and beyond

The phase 3 CLEAR study tested Lenvatinib plus Pembrolizumab vs. Lenvatinib plus Everolimus vs. Sunitinib monotherapy [44]. Lenvatinib plus Pembrolizumab had a PFS benefit over Sunitinib alone (median 23.3 vs. 9.2 months, HR 0.42) with sustained OS responses observed [45]. At a median follow up of 32.9 months, the CheckMate 9ER study of Nivolumab plus Cabozantinib vs. Sunitinib in 1st line demonstrated an OS and PFS advantage of 37.7 vs. 34.3 months (*p* = 0.0043) and 16.6 vs. 8.3 months (*p* < 0.0001), respectively [46]. This benefit conferred by ICIs was further supported by findings of the Keynote 426 study of Pembrolizumab plus Axitinib vs. Sunitinib monotherapy, which showed improved OS (median NR vs. 35.7 months (*p* = 0.0003)) and PFS (median 15.4 vs. 11.1 months (*p* < 0.0001)) [47]. These 3 combination studies denoted the impact of ICI for mRCC, and combination strategies are considered standard of care in the first line setting [20].

Triplet combination therapy in the COSMIC313 trial of 855 patients with intermediate–poor risk mRCC tested Cabozantinib plus Nivolumab plus Ipilimumab vs. Nivolumab plus Ipilimumab and showed improved PFS (median NR vs. 11.3 months, *p* = 0.013) whilst follow-up for OS continues [48]. Safety analysis showed higher grade 3–4 adverse events in the triplet arm (79% vs. 56%), predominantly liver enzyme elevations and hypertension, with drug discontinuation in 45% vs. 24% of patients, respectively [49]. A trial evaluating Atezolizumab with Bevacizumab versus Sunitinib did not demonstrate a PFS or OS benefit [50]. A comprehensive list of trials of TKI and ICI for mRCC are summarised in Table 1.

Whilst advancements in ICI and TKIs have changed the treatment landscape of RCC, treatment outcomes remain lacking with poor overall survival. In an immunogenic cancer such as RCC, novel strategies such as adoptive cell therapies represent an attractive approach. 

## 3. Non-Gene-Modified Cell Therapies

### 3.1. Cytokine Induced Killer Cells (CIKs)

#### 3.1.1. Biology and Background

CIKs are a heterogenous group of CD3^+^CD56^+^ natural killer (NK)-like cells and a subset of CD3^+^CD56^−^ T cells and CD3^−^CD56^+^ NK cells that are manufactured from peripheral or cord blood mononuclear cells that have been cultured with IFNγ, anti-CD3 monoclonal antibody and IL2 [51]. CIKs have been demonstrated in pre-clinical models to have anti-tumour activity that is MHC unrestricted and TCR independent [52,53]. They have a diverse TCR repertoire and have also been identified to express NK-like structures including natural killer group 2 member D (NKF2D) receptor, DNAX accessory molecule-1 (DNAM-1) and NKp30 [54]. In particular, engagement of the NKG2D receptor is recognised to mediate cytotoxicity against malignant cells [55].

#### 3.1.2. CIKs for mRCC

Early studies demonstrated the feasibility of CIKs in mRCC. A prospective study of autologous CIKs versus subcutaneous IL2 and IFNα-2a was conducted in 148 patients with metastatic ccRCC [56]. Autologous leukapheresis was collected from patients, and CIK cells were expanded in vitro for 14 days prior to infusion at a median cell dose of 97 x10^8^ total cells (range, 76–114 ×10^8^). ORR in the CIK arm vs. the IL2/IFNα arm was 53% vs. 27%; the 3-year PFS was 18% vs. 12% (*p* = 0.031), and the median OS was 46 vs. 19 months (*p* < 0.001). Building on this, a randomised study compared autologous CIKs vs. investigator choice of therapy in 20 patients following radical nephrectomy in stage I and II disease. In CIK-treated patients, CD3^+^, CD3^+^CD8^+^ and CD3^+^CD56^+^ populations in peripheral blood were increased 2 weeks post-infusion [57]. Whilst the sample size was small, median PFS (but not OS) was prolonged in the CIK arm (32.2 vs. 21.6 months, *p* = 0.032).

In a study of CIKs in 29 mRCC patients, an ORR of only 13.8% was observed (13.8%) and circulating MDSCs were reported to correlate with poor prognosis, highlighting the impact of the TME in mRCC [58].

A number of other mRCC CIK studies have shown minimal toxicity and CD3^+^CD56^+^ cell expansion in vivo, but without a clear message on clinical efficacy due to the small and heterogeneous nature of the studies [59,60].

#### 3.1.3. Combinatorial Approaches: CIKs + Dendritic Cells (DCs-CIKs)

Dendritic cell (DC) vaccination (and tumour antigen presentation) can potentially enhance CIK activation and cytotoxicity [61]. A randomised study of autologous DCs co-cultured with CIKs plus IFNα vs. no adjuvant therapy in patients post-surgical resection for RCC demonstrated a reduction in recurrence and metastatic disease (*p* < 0.01), with increased CD4^+^/CD8^+^ ratios and reduced CD4^+^/CD25^high^ populations [62]. A randomised trial of DC-CIK vs. no adjuvant treatment demonstrated a reduction in post-operative relapse (*p* = 0.0418) and higher 3-year DFS (96.7% vs. 57.7%) [63]. In 410 post-surgical mRCC patients, DC-CIK vs. IFNα demonstrated significantly improved 3-year OS (96% vs. 83% (*p* < 0.01), highlighting the potential of DC-CIK as an adjuvant treatment [64].

#### 3.1.4. Combinatorial Approaches: CIKs +/ Dendritic Cells +/ TKI +/ ICIs

Further studies have looked at strategies to bypass the immunosuppressive TME to further improve CIK and DC-CIK therapy. A phase 2 single arm study of 43 treatment-naïve or VEGFR-TKI r/r patients evaluated Axitinib plus autologous DC-CIK activated by low-dose Pembrolizumab in vitro and showed an ORR of 25.6% (95% CI 13.5–41.2%) with a median PFS of 14.7 months (95% CI 11.16–18.3). Inflammatory cell and cytokine signatures were increased after two and four cycles [65]. Pembrolizumab-activated DC-CIK products in combination with Pazopanib or Axitinib in 16 patients with locally advanced or complex disease in the neoadjuvant setting reduced tumour volume by a median of 42% (IQR 19.37–66.78%) [66]. In another study, CIKs plus anti-PD1 therapy was delivered every three weeks (up to a total of eight cycles) in 29 patients with r/r ccRCC [67]. The ORR was 41.4%, CR rate was 24.1%, median PFS was 15 months and median OS was 37 months, comparable to results reported in the CheckMate 025 study, albeit the numbers treated were smaller. Comparator studies are needed to evaluate clinical activity of this strategy. Pre-clinical work evaluating combination anti-CD40 and anti-CTLA-4 plus DC-CIKs have demonstrated efficacy in RCC cell lines [68,69], showing an advantage over monotherapy in renal cell line studies [70].

#### 3.1.5. Future Directions for CIK Therapy

The strength of CIKs lie in their relative ease of manufacturability and low toxicity. Combination with DC vaccines, TKI and/or ICIs are strategies to augment the cytotoxicity of CIK cells and improve efficacy by circumventing the immunosuppressive TME. An established international registry of CIK cells will ideally set a standard for future CIK studies [71].

### 3.2. Tumour Infiltrating Lymphocytes (TILs)

#### 3.2.1. Biology and Background

RCC immunogenicity is well described and underpins its responsiveness to immune-based therapies such as high-dose IL2 and ICIs. TILs are polyclonal, tumour-targeting T cells, expanded from patient tumour biopsies ex vivo for use as autologous products. In immunogenic tumours such as metastatic melanoma, overall response rates (ORR) are reported as 49–72%, CR is observed in 10–20%, with durable responses in 40% [72,73]. TILs are generally delivered following lymphodepleting chemotherapy (LD), most often comprising cyclophosphamide and fludarabine (Flu/Cy), with the goal of enhancing TIL expansion in vivo.

#### 3.2.2. TILs for mRCC

TILs are currently under investigation as a potential therapy for mRCC. An early phase 3 study of CD8^+^ TILs plus IL2 vs. IL2 alone for mRCC following radical nephrectomy demonstrated a high rate of unsuccessful manufacture, with low TIL viability and an ORR of 9.9% vs. 11.4%, respectively [74]. The study was closed early due to lack of efficacy. It is possible that TIL product quality following protracted ex vivo culture was to blame for the lack of efficacy. TIL manufacture has evolved substantially since the first clinical studies. Shortened TIL manufacture and rapid expansion protocol (REP) developed for melanoma has now been tested in mRCC using anti-CD3 antibody, high dose IL2 and irradiated feeder lymphocytes from healthy donors [75]. TIL expansion was successful, but products were functionally impaired, lacking cytotoxicity and cytokine secretion. It is possible that the repertoire of mRCC T cells lacked TSA/TAA recognition, or that product quality was impaired by the manufacture process. Other groups have similarly shown weaker antitumour activity and less cytokine polyfunctionality in mRCC compared to melanoma TILs [76].

A recent study compared the T-cell repertoire of pre-REP TILs versus REP TILs from RCC tumour samples by single cell RNAseq [77]. Results showed that REP predominantly expanded CD4+ T-cells, T-cell diversity was lost, and that exhausted T-cell clones did not expand. TIL manufacture using Dynabeads has demonstrated improved TIL expansion/functionality in vitro [78], and it is likely that future manufacturing advances will improve the feasibility and clinical impact of TILs for mRCC.

#### 3.2.3. Future Directions for TIL Therapy for mRCC

Improvements in manufacture to attain polyclonal tumour specific T-cell diversity and tumour reactivity will be key to the development of effective TIL therapy for mRCC.

## 4. Gene-Modified Adoptive Cell Therapies

Adoptive cell therapy harnesses the patient’s immune system to recognise tumour antigens and deliver an anti-tumour response. Challenges in the field however include antigen recognition, tumour escape due to the immunosuppressive tumour microenvironment and methods in manufacture that limit their potency and persistence. Gene modification of T and NK cells, such as with synthetic receptors in CAR-T and TCR-T cells, improves recognition of tumour-specific antigens (TSA) or tumour-associated antigens (TAA) and holds significant potential.

### 4.1. Chimeric Antigen Receptor T-Cells (CAR-Ts) and NK Cells (CAR-NKs)

#### 4.1.1. CAR-T Biology, Background, Targets

**Figure 2:**
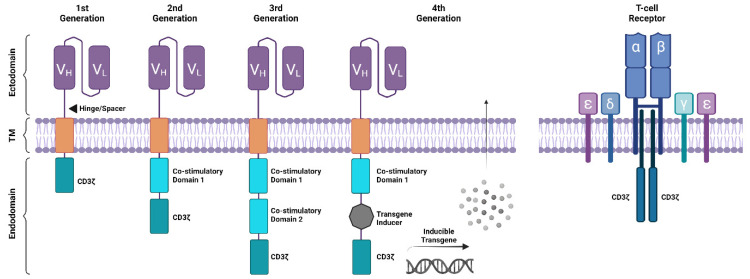
**Structure of Chimeric Antigen Receptors (CARs) and T-cell Receptors (TCRs).** The CAR structure is comprised of an ectodomain, transmembrane (TM) and endodomain region. The ectodomain includes a single-chain fragment variable (scFv) antigen-binding domain derived from the heavy (V_H_) and light (V_L_) chains of a monoclonal antibody (mAb), and a hinge/spacer commonly derived from CD8α/CD28/IgG to project the binding domain from the cell membrane provides flexibility in orientation. The transmembrane domain is commonly derived from CD8α/CD28 to anchor the CAR structure to the cell membrane. The endodomain houses an intracellular signalling domain derived from the TCRζ chain (CD3ζ), 1st generation CARs. Later generations incorporated a single T-cell co-stimulatory domain such as 41BB/CD28/ICOS/OX40 fused to CD3ζ to create more efficacious 2nd generation CARs. Later iterations are comprised of 3rd generation CARs that harbour two co-stimulatory domains fused to CD3ζ and 4th generation CARs which incorporate a module to permit transgene production following CAR activation. Additionally, 4th generations CARs (‘Armoured’ CARs) can be designed to induce the production of exogenous cytokines such as IL12/IL15 to augment CAR T-cell function/persistence or overcome a hostile tumour microenvironment (TME). Such transgenes can further include the production of ligands, enzymes, monoclonal antibodies and other immunomodulatory proteins. The structure of the TCR is composed of a αβ heterodimer paired with the invariant dimers CD3εδ, CD3εγ and CD3ζζ. The TCR permits recognition of antigenic peptides presented by major histocompatibility complex (MHC) molecules, and activation signals are propagated through the CD3 dimers.

CAR-T cells are T cells that have been engineered to express a chimeric antigen receptor (CAR) which is specific to an antigen on a tumour, enabling the cell to recognise and kill tumour cells. A CAR is comprised of an ectodomain and endodomain (Figure 2). The ectodomain is comprised of an antibody-derived single-chain variable fragment (scFv) antigen-binding domain connected to a spacer, transmembrane domain, and endodomain. In first-generation products, the endodomain was comprised of a sole CD3ζ-derived intracellular signalling domain, but later generation products now incorporate a co-stimulatory domain such as CD28 or 41BB, two co-stimulatory domains, or a single co-stimulatory domain with a transgene inducer module (Figure 2).

CAR-T for RCC is an area of active research with several promising CAR-T tumour targets identified. Here, we will focus on Carboxy-anhydrase-IX (CAIX), CD70, AXL, ROR2, and DNAJB8. At time of review, 10 phase I/II CAR-T clinical trials for RCC are recruiting (Table 2).

#### 4.1.2. Carboxy-Anhydrase-IX (CAIX) Biology

CAIX is an enzyme commonly overexpressed in hypoxic solid tumours, particularly in mRCC, and was one of the first antigens to be investigated as a potential CAR-T target for this condition [79].

#### 4.1.3. CAIX-Directed CAR-T Therapy

In a 12 patient phase I study, first-generation autologous anti-CAIX CAR-T cells for mRCC were infused daily (maximum ten doses) without LD [80,81]. No clinical responses were observed, but significant toxicity including deranged liver function was reported. The same group tested a self-inactivating bi-cistronic anti-CAIX-CAR-T with a CD28z endodomain, engineered to secrete anti-PD-L1 IgG1 or IgG4 as a payload at the RCC tumour site, to block PD-L1-mediated T-cell exhaustion [82]. In a humanised mouse model of CAIX^+^PDL1^+^ RCC, anti-CAIX-CAR secreting anti-PDL1 showed superior anti-tumour activity and reduced expression of the T-cell exhaustion markers than the control groups. This suggests that TME modulation may be critical to outcome in mRCC.

A range of anti-CAIX CAR-T constructs have been developed with the goal of improving RCC targeting and CAR-T persistence [83]. To overcome antigenic heterogeneity, dual-targeted fine-tuned immune-restoring (DFIR) CAR-T for ccRCC, targeting CAIX and CD70, and engineered for concurrent secretion of ICI is also undergoing pre-clinical testing [84].

Second-generation anti-CAIX CAR-T products have been tested in combination with Sunitinib [85] based on studies showing that Sunitinib increases IFNγ-secreting T-cells whilst reducing Tregs and MDSCs [86,87]. In a humanised mouse model of RCC, Sunitinib plus anti-CAIX CAR-T cells resulted in significantly reduced tumour burden compared to CAR-T and Sunitinib alone [85]. This requires further study.

#### 4.1.4. Allogeneic Approaches to CAIX-Directed CAR Therapy, including NK Cells

NK92 is an IL-2-dependent human-like NK cell line that can be used as starting material for allogeneic NK-CAR manufacture [88,89]. Third-generation anti-CAIX CAR-modified NK92 cells have demonstrated reduced tumour growth in immunodeficient RCC xenograft murine mouse models in combination with bortezomib [90]. Bortezomib is purported to enhance NK-cell-mediated anti-tumour effects [91].

#### 4.1.5. CD70 Biology

CD70 is a transmembrane glycoprotein that interacts with its receptor CD27 on T cells leading to effector and memory T-cell production [92]. CD70 is expressed on activated T- and B-lymphocytes and mature dendritic cells [93]. CD70 is also highly expressed in RCC making it an attractive therapeutic CAR-T target [94].

#### 4.1.6. CD70-Directed CAR-T Therapy

COBALT-RCC was a phase 1 multi-centre dose-escalation study of CTX130, an allogeneic CRISPR-Cas9-edited CAR-T product in advanced r/r ccRCC [95]. Patients had a median of three lines of prior treatment, and median CD70 expression on tumours was 100%. CAR-T expansion was seen across all dose levels and 3/14 patients had serious adverse events related to cytokine release syndrome (CRS). At ≥18 months of follow-up, 1/14 of patients had ongoing CR, 9/14 (69.2%) had SD, 4/14 (30.8%) had SD at 4 months, and the disease control rate was 76.9%.

The TRAVERSE study tested ALLO316, an allogeneic Transcription Activator-Like Effector Nuclease (TALEN) gene-edited anti-CD70 CAR-T product, in 17 patients with previously treated advanced or metastatic ccRCC [96]. TCR-alpha constant gene knockout was performed to reduce the risk of graft-versus-host-disease (GVHD), and CD52 knocked out to permit augmented lymphodepletion with ALLO627 (humanised anti-CD52 antibody) plus fludarabine and cyclophosphamide. A total of 11/17 patients (65%) developed CRS, with one (6%) grade 3 event. Disease control was achieved in 71% of patients, improving to 100% in those with confirmed CD70 expression (*n* = 9).

#### 4.1.7. Allogeneic Approaches to CD70-Directed CAR Therapy, including NK Cells

An allogeneic anti-CD70 CAR-NK construct named CAT-248, with CRISP/Cas9-mediated knockout of CD70 to avoid fratricide [97] and secreted IL15 to facilitate persistence, has shown activity in vitro and in a xenograft RCC model where a >99% reduction in tumour burden was observed vs. controls (*p* < 0.01) [98].

#### 4.1.8. AXL & ROR2 Biology

AXL is a tyrosine kinase receptor of the TAM kinase family and is overexpressed in many solid cancers, including RCC [99]. Along with its high-affinity ligand, the growth arrest-specific protein 6 (GAS6), AXL promotes tumour proliferation, survival, angiogenesis and invasion [100]. ROR2 is a member of the tyrosine kinase orphan receptor family that is important in embryologic development, albeit its role in adult tissue is unknown [101]. It has been found to be highly expressed in RCC and contributes to tumour growth, migration and invasiveness.

#### 4.1.9. AXL, ROR2-Directed CAR-T Therapy

A phase I/II two arm study is currently assessing safety and efficacy of the ROR2-targeting CAR-T product CCT301-59 and the AXL-targeting CAR-T product CCT301-38 in r/r metastatic RCC [102].

#### 4.1.10. DNAJB8 Biology

DNAJB8 is a cancer-testis antigen that is being investigated as another RCC antigen target, expressed in cancer stem-like cells and cancer initiating cells. It is known to contribute to tumorigenicity in RCC and osteosarcoma.

#### 4.1.11. DNAJB8-Directed CAR-T Therapy

Second-generation CAR-T incorporating the B10 binder (B10-CAR), designed to target an HLA-A*24:02/DNAJB8-derived peptide complex on RCC cells [103], shows antigen and HLA-dependent activation and IFNγ production with human RCC cell lines in vitro and a significant reduction in tumour burden in vivo (*p* < 0.01). Further studies are required to confirm that targeting cancer stem-like cell antigens is sufficient to treat entire tumours.

#### 4.1.12. P-MUC1C-ALLO1

This allogeneic CAR-T product targeting Mucin 1 (MUC1) cell surface associated C-Terminal antigen utilises a Cas-CLOVER gene editing system to knockout TCR and MHC class 1 proteins, and is being tested in a phase I trial of advanced/metastatic epithelial-derived tumours including RCC [104,105]. MUC1 is a transmembrane protein found on epithelial cells that usually has a protective function in normal cells but is commonly aberrantly glycosylated and commonly overexpressed in cancer, including RCC [106].

#### 4.1.13. C-Mesenchymal-Epithelial Transition Factor (c-met)

This tyrosine kinase has been shown to drive tumour migration, proliferation and invasion, and is overexpressed in 97% of papillary RCC (PRCC) but not normal renal tissue [107]. Third-generation c-met-targeting CAR-T, engineered with a fusion of CD28, 4-1BB and CD3ζ endodomains, was tested in an orthoptic RCC murine model, and suppressed tumour growth in 60% of mice, with histological demonstration of CAR+ and CD8+ T-cells in treated animals. This product was also tested in combination with Axitinib and a synergistic effect was reported [107].

#### 4.1.14. Epidermal Growth Factor (EGFR) Specific CAR-NK92

This construct has also been trialled in combination with Cabozantinib [108]. This product demonstrated antigen-dependent activation in vitro and in vivo, and Cabozantinib was shown to augment CAR-NK92 anti-tumour activity.

### 4.2. T-Cell Receptor Transduced T-Cells (TCR-Ts)

#### 4.2.1. TCR-T Biology, Background, Targets

Whilst CAR-T cells recognise antigens on the tumour cell surface, TCR-Ts are T cells with genetically engineered TCRs that recognise membrane and intracellular TAAs and TSAs presented by MHC molecules [109]. Binding of the TCR to recognised HLA-presented peptides causes a cytotoxic response and tumour killing, and the epitope density for activation is lower for TCR-Ts than what is observed for CAR-T therapy [110]. TCRs are HLA restricted, limiting the therapy to relatively common alleles such as HLA*02:01. During development of the product, strategies are required to avoid mispairing of exogenous TCR α and β to endogenous chains as well as methods to create high-affinity TCR products, although with caution due to risk of on-target off-tumour toxicities [111]. In this section, we focus on HERV-E as a viable TCR-T RCC tumour target.

#### 4.2.2. HERV-E-Directed TCR-T Therapy

Human endogenous retroviruses (HERVs) are remnants of exogenous retroviruses that have been integrated into the human genome [112]. They are usually silent, but aberrant expression has been identified in specific tumour types, including RCC, and can induce T-cell responses [14,113]. The transcripts CT-RCC-8 and CT-RCC-9, derived from a known HERV-E, are prevalent in ccRCC (but not normal) tissues. Other transcripts such as CT-RCC-ENV, which encodes the entire retroviral envelope gene, have also been identified [114]. Tumour-specific TCR-T engineered products have been developed for HERV-derived proteins, and a first-in-human phase 1 dose escalation trial of HERV-E TCR-T plus IL2 has been performed in 11 metastatic ccRCC patients [115]. Patients could tolerate HERV-E TCR-T doses of 5 × 10^7^/kg, and toxicities included grade 3–4 febrile neutropenia (57%), capillary leak syndrome (7%) and grade 2 skin rash (1/11). The median PFS was short at 62 days (IQR 31–90), but further data in the RCC TCR-T space is awaited.

## 5. Future Perspective of Cell Therapy against RCC

The development of adoptive cell therapies against RCC remains in the early stages but is progressively building momentum. Several hurdles remain in the development of such cell therapies for solid tumours. Firstly, a lack of heterogeneously expressed TSAs with limited off-target toxicities remains a critical problem. CAR-T tumour targeting candidates for RCC including CAIX have demonstrated significant therapy limiting off-target liver toxicity [81] and off-target CD70 expression on activated T- and B-lymphocytes, and mature dendritic cells are a concern [93]. Whilst lack of tumour heterogeneity can only be addressed through multi-antigen targeting, bypassing off-target toxicity can be achieved through next-generation engineering approaches through ‘AND’ and ‘AND-NOT’ logic gates. ‘AND’ gates permit the identification of TSA#1 which drives CAR transgene expression to target TSA#2. ‘AND-NOT’ gates enable the inhibition of CAR activation upon TSA binding following binding to a normal antigen [116,117]. Safety can further be managed through designed ‘ON/OFF’ switches where CAR expression can be held in an ‘OFF’ state, and removal of inhibition regulated by small molecules or hypoxia/proteases within the TME is required for CAR activation [116,118,119]. Similarly, CD70 target expression on T cells itself can result in CAR-T fratricide where gene-editing mediated knockdown of CD70 [120] would be required but poses further safety considerations pertaining to gene-editing-related off-target genome toxicity. Ultimately, there is a greater need to identify improved neoantigens for the progress of both CAR and TCR-T therapies which rely on the optimisation of advanced computational and functional screening pipelines [121].

Secondary and tertiary hurdles are formed by maximising T-cell trafficking within solid tumours and maintaining persistence by overcoming the immunosuppressive TME. Engineering strategies to drive expression of chemokines receptors such as CXCR1/2/4 have shown promising results in enhancing tumour trafficking [122,123]. Enhanced trafficking was also observed by targeting cancer-associated stromal cells (CASCs) through the fibroblast activation protein (FAP), a protease with selective expression on CASCs in a CAR-dependent manner [124]. Additionally, evaluating routes of administration beyond systemic infusion of adoptive cells to enhance trafficking is worthy of further investigation. Similarly, other engineering strategies to armour adoptive cells therapies against the TME include T cells redirected for universal cytokine-mediated killing (TRUCKs) to secrete cytokines such as IL-12 to overcome TREG- and MDSCs-regulated immunosuppression and stimulate an innate immune response [125]. Other cytokines, including IL-15, IL-7 and IL-18, have also been assessed to enhance T-cell responses [126,127,128]. Further engineering methods include the use of dominant negative receptors or the use of switch receptors where an ectodomain binds to an immunosuppressive target fused to a T-cell stimulatory endodomain. Both have been assessed in the context of TGF-β, a cytokine that can drive immunosuppressive M2 macrophage polarisation and TREG differentiation. Use of a dominant negative TGF-β receptor [129] and a TGF-βR2:41BB switch receptor have been shown to enhance CAR-driven anti-tumour responses [130]. Additional strategies can include the combinatorial use of antibody-driven PD-1/CTLA-4 immune checkpoint inhibition [131], engineering methods via gene-editing-based knockout of ICIs on T cells such as PD-1 [132] and CAR-T-driven secretion of anti-PD-L1 antibodies in the TME [82,133]. Lastly, development of CAR transduced macrophages (CAR-M) are also being explored for their superior tumour homing capabilities, tumour cell phagocytosis and induction of surrounding immune cells [134].

It is apparent that treatment of solid tumours with adoptive cell therapies will require combinatory treatment approaches with next-generation cell engineering modules for enhanced treatment outcomes. The ideal therapy combinations and engineering designs remain undefined, and many such strategies remain unexplored in the context of RCC, where evaluation of such methods will be critical to success.

## 6. Conclusions

RCC is an immunogenic cancer, but despite this, improving treatment outcomes in the r/r setting continues to be challenging. Adoptive cell therapies hold significant promise, but many products remain in their infancy and are predominantly being tested in the preclinical space. Experimental work has identified multiple potential antigenic targets and therapeutic combinations with TKIs and ICIs, but the optimal target antigen remains unknown. Newer-generation armoured CAR products or combinatory treatments will also likely need to be utilised to bypass the TME. Ultimately, cell therapies for RCC will require testing in large, randomised controlled trials with appropriate controls that help us understand how they fit into the current treatment paradigms in the r/r RCC space.

## Figures and Tables

**Table 1:** **Registered tyrosine kinase inhibitor and immune checkpoint inhibitor combination therapies for Renal Cell Carcinoma in Phase 3 including FDA approved therapies.** Key: RCC—Renal Cell Carcinoma, mRCC—metastatic Renal Cell Carcinoma, ccRCC—clear cell Renal Cell Carcinoma, PRCC—Papillary Renal Cell Carcinoma, TKI—tyrosine kinase inhibitor, PD-1—programmed death-1, IL—interleukin, VEGF—vascular endothelial growth factor, ICI—immune checkpoint inhibitor, mTOR—Mammalian target of rapamycin, CTLA—Cytotoxic T-lymphocyte associated protein, IDO1—Indoleamine 2,3-dioxygenase 1, HIF—Hypoxia-Inducible Factor. (This list was composed using the registered trial candidates on ClinicalTrial.gov, WHO Clinical Trials Registry and the Food and Drug Administration database.)

Clinical Trial	Primary Endpoint	Treatment Description	Condition	Sponsor	Phase	Status
FDA approved combination therapies						
FDA approved combination of Nivolumab plus Ipilimumab in April 2018						
NCT02231749 (CheckMate 214)	ORR PFS OS	(PD-1 inhibitor+ CTLA-4 inhibitor vs. TKI) Nivolumab + Ipilimumab vs. Sunitinib monotherapy	First-line treatment of intermediate-/poor-risk advanced RCC	Bristol-Myers Squibb	3	Active, not recruiting
FDA approved combination of Pembrolizumab plus Axitinib in April 2019						
NCT02853331 (Keynote 426)	PFS OS	(PD-1 inhibitor + TKI vs. TKI monotherapy) Pembrolizumab + Axitinib vs. Sunitinib monotherapy	First-line treatment of advanced ccRCC	Merck Sharp & Dohme LLC	3	Active, not recruiting
FDA approved combination of Avelumab plus Axitinib in May 2019						
NCT02684006 (Javelin 101)	PFS OS	(PD-1 inhibitor + TKI vs. TKI monotherapy) Avelumab + Axitinib and of Sunitinib monotherapy	First-line treatment in patients with advanced RCC	Pfizer	3	Active, not recruiting
FDA approved combination of Nivolumab plus Cabozantinib Jan 2021						
NCT03141177 (CheckMate 9ER)	PFS	(PD-1 inhibitor +TKI vs. TKI) Nivolumab + Cabozantinib vs. Sunitinib	First-line treatment of advanced or mRCC	Bristol-Myers Squibb	3	Active, not recruiting
FDA approved combination of Lenvatinib plus Pembrolizumab in August 2021						
NCT02811861 (CLEAR)	PFS	(TKI+ mTOR inhibitor or PD-1 inhibitor vs. TKI monotherapy) Lenvatinib + Everolimus or Pembrolizumab vs. Sunitinib monotherapy	First-line treatment of advanced RCC	Eisai Inc.	3	Active, not recruiting
Other TKI and ICI combination therapies						
NCT04338269 CONTACT-03	PFS OS	(ICI+TKI) vs. TKI monotherapy Atezolizumab + Cabozantinib vs. Cabozantinib monotherapy	Advanced RCC	Hoffmann-La Roche	3	Active, not recruiting
NCT03138512 (CheckMate 914)	DFS	(PD-1 inhibitor monotherapy vs. PD-1 inhibtor+ CTLA-4 inhibitor vs. placebo) Nivolumab, Nivolumab + Ipilimumab and Placebo	Localized kidney cancer with removal of part of a kidney	Bristol-Myers Squibb	3	Completed
NCT03288532 (RAMPART)	DFS OS	(PD-1 inhibitor monotherapy vs. PD-1 inhibitor monotherapy+ CTLA-4 inhibitor) Durvalumab monotherapy or Durvalumab + Tremelimumab	Resected primary RCC at high or intermediate risk of relapse	University College London	3	Recruiting
NCT05239728	DFS	(HIF-2α inhibitor+PD-1 inhibitor vs. placebo+PD-1 inhibitor) Belzutifan + Pembrolizumab vs. placebo +Pembrolizumab	Adjuvant treatment of ccRCC post nephrectomy	Merck Sharp & Dohme LLC	3	Recruiting
NCT04394975 (JS001-036-III-RCC)	PFS	(PD-1 inhibitor +TKI vs. TKI monotherapy) Toripalimab + with Axitinib vs. Sunitinib monotherapy	First-line therapy for advanced RCC	Shanghai Junshi Bioscience Co., Ltd.	3	Active, not recruiting
NCT03937219 (COSMIC-313)	PFS	(TKI+PD-1 inhibitor+ CTLA-4 inhibitor vs. PD-1 inhibitor+ CTLA-4 inhibitor +matched placebo) Cabozantinib + Nivolumab + Ipilimumab vs. Nivolumab and Ipilimumab + matched placebo	Intermediate- or poor-risk advanced or mRCC	Exelixis	3	Active, not recruiting
NCT04736706 NCT05899049 or MK-6482-012 (China extension study) (LIFESPARK-012)	PFS OS	(PD-1 inhibitor +HIF-2α inhibitor+ TKI or PD-1 inhibitor/CTLA-4 inhibitor +TKI vs. PD-1 inhibitor+TKI) Pembrolizumab + Belzutifan + Lenvatinib or Pembrolizumab/Quavonlimab + Lenvatinib vs. Pembrolizumab + Lenvatinib	First-line treatment in participants with advanced ccRCC	Merck Sharp & Dohme LLC	3	Recruiting
NCT03873402 CA209-8Y8	PFS ORR	(PD-1 inhibitor + CTLA-4 inhibitor vs. PD-1 inhibitor monotherapy) Nivolumab + Ipilimumab vs. Nivolumab monotherapy	Untreated kidney cancer that has spread	Bristol-Myers Squibb	3b	Active, not recruiting
NCT03793166 (PDIGREE)	OS	(PD-1 inhibitor+ CTLA-4 inhibitor followed by PD-1 inhibitor or PD-1 inhibitor + TKI) Nivolumab and Ipilimumab Followed by Nivolumab or Nivolumab with Cabozantinib	Advanced kidney cancer	National Cancer Institute (NCI)	3	Recruiting
NCT05219318	DP	PD-1/PD-L1 ICI + VEGFR-TKI	First-line ICI for mRCC	University Hospital, Bordeaux	3	Recruiting
NCT03260894 (KEYNOTE-679/ECHO-302)	ORR	(PD-1 inhibitor + IDO1 selective inhibitor vs. TKI) Pembrolizumab+Epacadostat vs. Sunitinib or Pazopanib	First-line treatment for mRCC	Incyte Corporation	3	Active, not recruiting
NCT05678673	PFS ORR	(TKI+ PD-1 inhibitor vs. TKI) XL092 + Nivolumab vs. Sunitinib	Unresectable, locally advanced or metastatic nccRCC who have not received prior systemic anticancer therapy	Exelixis	3	Recruiting
NCT05043090	PFS	Savolitinib + Durvalumab vs. Sunitinib	MET-driven unresectable and locally advanced or metastatic PRCC	AstraZeneca	3	Recruiting
NCT01668784 (CheckMate 025)	OS	(PD-1 inhibitor vs. mTOR inhibitor) Nivolumab vs. Everolimus	Advanced or mRCC after failure of one or two regimens of anti-angiogenic therapy	Bristol-Myers Squibb	3	Completed
NCT01865747 (METEOR)	PFS	(TKI vs. mTOR inhibitor) Cabozantinib (XL184) vs. Everolimus (Afinitor)	mRCC after prior VEGF-targeted therapy	Exelixis	3	Completed
NCT01030783	PFS	(TKI vs. TKI) Tivozanib vs. Sorafenib	First targeted therapy in recurrent or mRCC	AVEO Pharmaceuticals, Inc.	3	Completed
NCT02420821 (IMmotion 151)	DP PFS OS	(ICI+VEGF inhibitor vs. TKI) Atezolizumab + Bevacizumab vs. Sunitinib	Untreated advanced RCC	Hoffmann-La Roche	3	Completed
CA045002 NCT03729245 (PIVOT 09)	ORR OS	(IL-2 pro-drug + PD-1 inhibitor vs. TKI monotherapy) Bempegaldesleukin+ Nivolumab vs. (Sunitinib or Cabozantinib)	Previously untreated advanced RCC	Nektar Therapeutics	3	Terminated
NCT04523272 (TQB2450-III-07)	PFS	(PD-1 inhibitor+ TKI vs. TKI) TQB2450 + Anlotinib vs. Sunitinib	Advanced RCC	Chia Tai Tianqing Pharmaceutical Group Co., Ltd.	3	Unknown

**Table 2:** **Registered Chimeric Antigen Receptor (CAR) therapy clinical trials for Renal Cell Carcinoma (RCC). Key:** RCC—Renal Cell Carcinoma, mRCC—metastatic Renal Cell Carcinoma, ccRCC—clear cell Renal Cell Carcinoma, CAR—Chimeric Antigen Receptor, PBL—Peripheral Blood Lymphocytes (This list was composed using the registered trial candidates on ClinicalTrial.gov and the WHO Clinical Trials Registry).

Clinical Trial	Primary Endpoint	Description	Condition	Company	Phase	Target Antigen	Status
**CAR T therapy targeting CD70**							
NCT04696731	DLT	Allogeneic ALLO-316	Advanced or metastatic ccRCC	Allogene Therapeutics	1	CD70	Recruiting
NCT04438083	ORR DLT	CTX130	Relapsed or refractory RCC	CRISPR Therapeutics AG	1	CD70	Active, not recruiting
NCT06010875 NCT05468190	Safety evaluation and tolerability	CD70-targeting CAR-T cells	Advanced/metastatic solid tumours	Chongqing Precision Biotech Co., Ltd.	1	CD70	Recruiting
NCT05420545 NCT05420519	Safety evaluation and tolerability	CAR-T	Advanced/solid tumours including RCC	Chongqing Precision Biotech Co., Ltd.	1	CD70	Recruiting
NCT05518253	Safety evaluation and tolerability	CAR-T	Advanced/solid tumours including RCC	Weijia Fang, MD	1	CD70	Recruiting
NCT05795595	DLT ORR	Allogeneic CTX131	Relapsed or refractory solid tumours including ccRCC	CRISPR Therapeutics AG	1/2	CD70	Recruiting
**CAR NK cell therapy**							
NCT05703854	Safety evaluation and optimal cell dose	CAR.70-engineered IL15-transduced Cord Blood-derived NK Cells in Conjunction with Lymphodepleting Chemotherapy	Advanced RCC	M.D. Anderson Cancer Center	1/2	CD70	Recruiting
**CAR PBL therapy**							
NCT02830724	Safety evaluation RR	PBL Transduced with a CD70-Binding CAR	RCC	National Cancer Institute (NCI)	1/2	CD70	Recruiting
**CAR T therapy with different target antigen**							
NCT03393936	Safety evaluation ORR	Autologous CCT301-38 or CCT 301-59 T cells	Relapsed and refractory stage IV mRCC	Shanghai PerHum Therapeutics Co., Ltd.	1/2	ROR2	Unknown
NCT01218867	Safety evaluation	Anti-VEGFR2 gene modified tumour white blood cells	mRCC	National Cancer Institute (NCI)	1/2	VEGFR2	Terminated
NCT03638206	Safety evaluation	Autologous CAR-T/TCR-T cell immunotherapy	Different malignancies including RCC	Shenzhen BinDeBio Ltd.	1/2	c-MET	Unknown
NCT04969354	Safety evaluation ORR CR PR SD PD	CAR T cells targeting CAIX	Advanced RCC	The Affiliated Hospital of Xuzhou Medical University	1	CAIX	Recruiting
NCT05239143	MTD RP2D DLT ORR	P-MUC1C-ALLO1 allogeneic CAR-T cells	Advanced or metastatic solid tumours	Poseida Therapeutics, Inc.	1	Mucin 1 cell surface-associated C-terminal	Recruiting
NCT05672459	ORR	Autologous HLA-G- Targeted CAR-T Cells IVS-3001	Previously treated advanced HLA-G-positive solid tumours	M.D. Anderson Cancer Center	1/2	Human leukocyte antigen (HLA-G)	Recruiting

## Data Availability

No data were generated in the review. The data summarized in Table 1 and Table 2 were sourced from cited papers.
